# Researchers Add to Proteomics Toolbox

**DOI:** 10.1371/journal.pbio.0020038

**Published:** 2004-01-20

Genes use a simple language—written in the molecules of DNA—to build thousands of proteins in a dizzying variety of sizes and shapes. With only four different nucleotide building blocks, DNA codes for the 20 different amino acids (each with their own structures and properties) that provide the foundation for the enormous diversity of protein form and function. This diversity makes the systematic study of all the proteins of a given organism (called proteomics), a challenging enterprise.

Interactions between proteins underlie nearly every fundamental process within the cell. They can form higher-order multiprotein complexes like those involved in transcription and replication, help transport proteins to their proper location in the cell, and participate in signaling pathways. Because of their importance, disruption of these interactions can have disastrous consequences. For example, the loss of the ability of a normal cellular protein called Src to bind to certain other proteins can be associated with cancer progression. The determinants of these interactions are poorly understood, but in many cases these interactions are mediated by small pieces of the proteins, which are called peptides. Peptides serve as the starting point for the novel strategy reported in this issue.

Gianni Cesareni and colleagues have added to the repertoire of proteomic analysis by devising a global strategy to investigate protein–protein interactions on an organismal level using yeast as a model organism. The authors select a protein of interest from yeast, which can be thought of as the “bait” for which they wish to identify protein-binding partners. They start by looking at a number of different previously identified peptides that bind the bait protein. Commonalities between the sequences of these peptides form the “consensus” binding sequence, a base framework of protein sequence from which many possible variations can be derived. Since the protein sequences of all proteins (the proteome) in yeast can be deduced from the sequenced genome, the authors can scan the proteome for proteins that contain the consensus, or a closely related, sequence. These proteins could potentially bind the bait protein. Peptide sequences from these identified proteins are synthesized chemically and arrayed on a membrane, which is bathed in a solution containing the bait protein. After washing off the excess bait protein, they can figure out where it remains on the membrane and therefore tell which peptides the bait protein has bound. The proteins corresponding to these peptides are candidate binding proteins that are validated by further experimentation.

The protein–protein interactions identified by this approach can be used to extend the network of known interactions in the proteome. This will enable researchers to draw functional linkages between proteins, whether they are involved in a basic biological process or in human disease. By examining whole families of proteins, it may also aid in elucidating the underlying determinants of binding specificity, which would provide clues to the biomechanisms underlying cell processes. These insights could lead to methods for manipulating these interactions, perhaps even in cases of human disease, as in the case of Src and cancer. This approach can readily be applied to the proteomes of more complex organisms like humans and adds to the growing number of experimental strategies available to researchers in proteomics.

**Figure:**
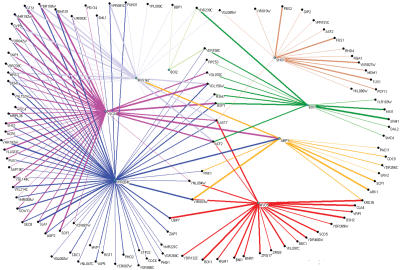
Protein interaction network

